# Social and Cultural Barriers to Diabetes Prevention in Oklahoma American Indian Women

**Published:** 2004-03-15

**Authors:** Kathryn S. Keim, Christopher Taylor, Alicia Sparrer, Jean Van Delinder, Stephany Parker

**Affiliations:** Department of Nutritional Sciences, Oklahoma State University; Department of Nutritional Services, Oklahoma State University; Harris Methodist Fort Worth Hospital; Department of Sociology, Oklahoma State University; Cooperative Extension Service, Oklahoma State University

## Abstract

**Introduction:**

The prevalence of diabetes is disproportionately higher among minority populations, especially American Indians. Prevention or delay of diabetes in this population would improve quality of life and reduce health care costs. Identifying cultural definitions of health and diabetes is critically important to developing effective diabetes prevention programs.

**Methods:**

In-home qualitative interviews were conducted with 79 American Indian women from 3 tribal clinics in northeast Oklahoma to identify a cultural definition of health and diabetes. Grounded theory was used to analyze verbatim transcripts.

**Results:**

The women interviewed defined health in terms of physical functionality and absence of disease, with family members and friends serving as treatment promoters. Conversely, the women considered their overall health to be a personal issue addressed individually without burdening others. The women presented a fatalistic view of diabetes, regarding the disease as an inevitable event that destroys health and ultimately results in death.

**Conclusion:**

Further understanding of the perceptions of health in at-risk populations will aid in developing diabetes prevention programs.

## Introduction

The American Indian people and culture have sustained serious hardships throughout the last 2 centuries; their greatest struggle, however, may be impending. The rate of diabetes is disproportionately higher in minority populations, especially the American Indian population (1-4). Indian Health Service (IHS) national outpatient data indicate that the age-adjusted prevalence rate of diabetes among American Indians is an estimated 88.7 per 1000 for individuals older than 15 years (5). In the Oklahoma City area, the largest of IHS areas, the age-adjusted prevalence rate of diabetes is 60 per 1000 individuals (3), indicating that American Indians are 2.43 times more likely to have diabetes than the general population at 39 per 1000 individuals (6). Furthermore, national data indicate age-adjusted prevalence rates are greater for American Indian women (12.0%) compared to American Indian men (9.7%) (3). Lee et al observed in an Oklahoma sample that 38% of American Indian men had diabetes compared with 42% of American Indian women (7).

Diabetes is a multifaceted disease that is reaching epidemic proportions in the American Indian community (1). If diabetes could be prevented or delayed in this population, the benefits in quality of life and health care cost savings would be considerable. Rhoades et al estimated 882 years of productive life lost due to diabetes mellitus over a 3-year period among American Indians receiving health care services from IHS (8). Diabetes results in compromises to longevity and quality of life and in economic disadvantages. Health care costs for treatment of non-American Indian patients with diabetes in 1994 were 2.4 times greater than non-American Indian controls, with long-term complications accounting for 38% of the costs (9). Through a Monte Carlo study based on American patients with diabetes, intensive blood sugar control was estimated to produce a 3% reduction in health care costs over 30 years (10). Additionally, Oklahoma Behavioral Risk Factor Surveillance System data demonstrated a significantly greater number of days of disability and poor physical health for patients with diabetes compared to control subjects without diabetes (11). These data have obvious ramifications for workplace productivity. Success at delaying or preventing the onset of diabetes will reduce the costs of diabetes treatment and prolong an individual's potential to be a contributing member of the economy.

A greater understanding of American Indian perceptions of health and diabetes is paramount to the success of diabetes prevention programs among these populations (12-15). Perceptions of the inevitability of diabetes within the reservation environment have been reported (16-18). Perceptions of health among American Indian elders in an urban setting have also been presented (19). Data is lacking on the relationship of diabetes to health and the social environment as well as the perception of the feasibility of diabetes prevention. This study used in-depth qualitative interviews to ascertain a cultural definition of health and diabetes from American Indian women residing outside a reservation setting. The information learned will be used to plan culturally appropriate nutrition education and health promotion programs aimed at preventing or delaying the onset of diabetes among American Indians in Oklahoma.

## Methods

The data contained herein represent a portion of a larger study that involved a series of 3 sessions with each study participant. The first session included demonstration of informed consent, completion of a demographic questionnaire and a rank-order assessment of life concerns, and training for a 4-day weighed-food record collection. During the second interview, the participants responded verbally to questions from the *Cultural Structure of Health and Diabetes* questioning guide (Table 1) and completed a free food sort of previously determined most commonly consumed foods. The food sort allowed participants to group foods based on their own classifications. The final session included a weight valuation interview to identify the cultural perceptions of body image and a trichotomous food sort of the most commonly consumed foods. In this sort, participants sorted food into groups based on their perceptions of health value and fat and sugar content. The research protocol was reviewed and approved by the Institutional Review Board at Oklahoma State University and the executive counsels for the cooperating tribes.

### Interviewer training

Five female American Indian interviewers were hired to conduct the in-depth interviews. Each interviewer completed a one-day course on subject recruitment, interview structure, data collection techniques, and response recording. The training consisted of equipment usage, essential techniques of qualitative interviewing (listening and directive questioning skills, for example) and the logistics of the qualitative interviews. The interviewers were compensated $100 for training and $125 for each participant who completed 3 interviews.

### Participants

Women of at least one quarter American Indian blood, between ages 18 and 65 years, who were not pregnant or lactating, were eligible for the study. A diagnosis of chronic disease, including diabetes, did not prevent inclusion; however, women diagnosed with chronic diseases that have an impact on appetite (including women receiving cancer treatment) were excluded from the study. Women were recruited proportionately from tribal health clinics in northeast Oklahoma using a non-probablility sampling design. To increase participation rates, women who successfully completed the interview process received $125.

Key informants and the American Indian interviewers at each of the clinics recruited potential subjects for the research study. Articles were published in tribal newsletters and newspapers to promote the study. Women interested in participating were referred to one of the interviewers to receive more information, determine eligibility, and schedule interviews. Additional subjects were recruited from tribal diabetes education programs and the 3 tribal general health clinics.

### Data collection and analysis

This study reports results of the interviews during the second session, in which participants responded verbally to questions from the *Cultural Structure of Health and Diabetes* questioning guide (Table 1). Questions from previous research (20) were modified to identify cultural perceptions of health and diabetes. Questions focused on areas of interest that were consistent with the objectives of the study, such as perceived causes, treatments, and efficacy of diabetes prevention behaviors. Key informants within each clinic reviewed the questions for cultural sensitivity prior to their administration. Results based on each interviewer's session with the first participant served as a pilot; responses were analyzed as the data became available and appropriate changes were made to the questioning guide.

Two researchers analyzed the verbatim transcripts from the audiotapes during data collection. Grounded theory guided analysis of the transcripts (21). An initial list of code words was derived from recurring themes in the transcripts (Table 2). Then, key concepts or recurring themes derived from the qualitative interviews were integrated into the questioning guide using the method of constant comparisons. The transcripts were reviewed throughout the interviewing process. Code word definitions were drafted to encompass the meaning of text segments. When new themes recurred in the transcripts, they were either assigned a new code word or a subcategory of an existing code word. Furthermore, the questioning guide was modified to capture more detail about the emerging themes. Text segments were coded with the corresponding code words using Ethnograph (version 5.04, Qualis Research Associates, Denver, CO). Following open coding, axial coding was used to identify subcategories with code words (21). The final step, selective coding, provided the means to assess the relationship among constructs and to assess how concepts were related to their constructs to establish an overall phenomenon.

## Results

Eighty-one American Indian women completed the qualitative interviews. Two transcripts were not available because of technical failure of the recording devices, resulting in 79 usable interviews. Demographic characteristics of the sample are provided in Table 3. The mean age of the women was 43 ± 11 years while mean degree of American Indian blood was 65%. Though the sample was collected from 3 tribal health clinics, 16 different tribal affiliations were reported, making analysis by tribe impractical. Of the 79 women, 26 (33%) reported a previous clinical diagnosis of diabetes. Approximately 70% reported education beyond high school; however, 72% indicated an annual household income of less than $25,000.

Twenty-nine unique code words were developed during the open coding of the transcripts (Table 2). Text segments coded for each code word were then analyzed to establish subcategories and relations among code words. The results of the analysis for code words associated with health and diabetes are presented below.

### Cultural definition of health

The American Indian women who took part in this study defined health predominantly in terms of lifestyle behaviors. Individuals performing positive behaviors — such as consuming a "healthy" diet, exercising, and not smoking — were considered healthier than those who did not. Being overweight was also considered to reflect negatively on health status.

Health was also defined in terms of the presence or absence of disease. For example, when individuals were asked to define their current health, they sometimes mentioned the presence or absence of several chronic conditions, including arthritis, diabetes, heart disease, and cancer. In the absence of a chronic disease, individuals considered themselves to be healthy. Even if clinically diagnosed with disease, individuals did not perceive diminished health until there was a physical feeling of illness. Until an individual perceived a feeling of illness, they considered their health to be satisfactory. One woman said, "I haven't been throwing-up sick in years, but a little cold here and there." This was especially true of diabetes, as the women interviewed did not consider the disease to be severe until it was manifested through long-term complications.

Another indicator of health status was defined through physical functionality. The women considered poor health to be an impairment of one's ability to perform daily tasks: "Oh, my current health. I feel like I'm pretty healthy. I can still lift up things and get around." The women viewed being healthy as having the capacity and energy to perform daily tasks and other activities. However, certain accommodations were made for age. Furthermore, the women expected health to decline with age; many defined their health status according to expectations for their current age. One woman described feeling "not too good about my health and myself. It seems like I've been more tired. But I guess that's just this age."

### Cultural definition of diabetes

Diabetes was defined most commonly in terms of long-term complications, which were often tied to fear and concern. The most frequently noted complication was amputation, expressed by one woman as "becoming a member of the stub club." Some women were confused about diabetes and its symptomology and long-term complications. Many women were unclear about long-term complications; some women said that dialysis and blindness were symptoms of diabetes. Confusion about hyperglycemia and hypoglycemia — and which one indicated diabetes — also existed. The women expressed the belief that hypoglycemia is an early symptom of diabetes that later converts to hyperglycemia.

Similarly, others expressed a fear of diabetes, calling it a "scary disease." Diabetes was portrayed as devastating. As one participant said, "It ruins your health, and ultimately it will kill you." Furthermore, diabetes was considered a malicious disease. One woman stated: "Diabetes is scary. It's a scary process. It's demeaning. I think it is a very, very cruel breakdown of your system." The perception existed that a body being "out of balance" causes diabetes, and an error in the inner workings of the body results in a blood sugar imbalance.

"Fatalism" (16) toward diabetes and its complications was a strong theme among the women. One woman said, "I knew it was going to happen, but when it did happen, it was a surprise to me. And I felt like I was doomed." The women interviewed expressed the concern that being of American Indian descent leads to a belief in increased susceptibility to diabetes as well as a belief in the inevitability of getting diabetes. Furthermore, the women feared having diabetes for an extended period of time without being diagnosed. The American Indian social network also fostered apprehension about diabetes, as most of the interview participants knew someone with the disease.

Another prominent concern among the American Indian women was the possibility of their own or a family member's diagnosis of diabetes. Interestingly, the women were more concerned about their children being diagnosed with diabetes than about their own possible diagnosis. Their statements about children being at risk reflected an overall concern for children developing diabetes. The women were also concerned about other family members, including siblings, spouses, and parents.

The women expressed the idea that after an individual is diagnosed with diabetes, his or her lifestyle behaviors must change. Diabetes was perceived to require thorough, demanding care. Appropriate care involved eating right, taking medicine, and doing "what the doctor tells you to do." The women regarded diabetes care and behavior change as solely the responsibility of the individual.

### Diabetes prevention

When asked if it was possible to prevent diabetes, many of the women responded in terms of personal behaviors that may prevent or help delay the onset of diabetes. These responses centered on changing behaviors that cause diabetes, such as eating a poor diet and not exercising. To explore those responses, we asked further questions about when potential preventative behaviors should begin, and a portion of the respondents indicated the need to reach young children. Other participants with a more fatalistic view of diabetes suggested that diabetes was inevitable in individuals with a strong family history of the disease.

### Barriers to diabetes prevention and treatment

Some interview participants indicated that frequent visits to their health care professionals represented an appropriate method of diabetes prevention. Furthermore, the women perceived diabetes screening as a method of diabetes prevention in the absence of changing lifestyle factors. Issues of denial and avoidance of diagnosis were also strong, providing an additional challenge to diabetes prevention and treatment. Despite efforts to increase public awareness and opportunities for diabetes screening, women still avoided screening. Because an individual was considered to be in good health in the absence of physically feeling ill or the clinical diagnosis of a chronic disease, avoiding a visit to a health care professional (thus avoiding a screening) freed the individual from diagnosis and evaded the need for self-care — despite a personal suspicion of having the disease. One woman mentioned "[t]here might be a tendency for people to suspect it but not want to have it confirmed maybe." In such situations, care for diabetes is delayed and the likelihood of long-term complications increases.

Furthermore, individuals did not express personal concern about diabetes until they were themselves facing diagnosis. If a positive diagnosis was made, those women expressed a strong sense of denial. One participant mentioned a family member who was "in denial, and won't go to the doctor, and then it gets worse, and then they'll go after it starts getting too bad." Individuals often postponed care until they perceived a physical ailment, likely indicative of long-term complications.

### Supporting social structure

The women mentioned many sources of social support and information. They cited health care professionals as only one of many sources of information about health and diabetes. Community and family members served as considerable sources of both information and misinformation. Misconceptions ranged from the idea that individuals with diabetes are forced into strict dietary modifications with a complete absence of sugar to the idea that diabetes can be "gotten rid of, if you take care [of yourself]." The women obtained much information about diabetes prevention, symptoms, and treatment from discussions with — or observation of the treatments received by — immediate or extended family and friends. Shared knowledge within these circles does not reflect the current state of diabetes care, but defers to an older pedagogy of diabetes care.

In addition to serving as sources of information, families were portrayed as mediators of health self-care. Many self-care concerns are rooted in the women's family caregiver roles, especially as gatekeepers of healthy meals. Their roles are challenged by having to make personal lifestyle behavior changes. For example, American Indian women are responsible for providing meals that satisfy the entire family. If their health requires dietary changes, they find it unacceptable to put their needs above the wants or needs of the family unit, greatly reducing the likelihood of behavior modification.

When asked how the family could aid in diabetes prevention efforts, familial and parental support was most commonly reported. Family discussions about health and diabetes as well as family attendance of educational sessions were indicated as methods of family involvement in diabetes prevention. However, one woman indicated that when she suspected she might have diabetes, her family discouraged screening because they thought it unlikely she would be diagnosed with the condition. This demonstrates both the positive and negative social environment affecting diabetes prevention and treatment.

## Discussion

The qualitative method used in our study demonstrates an attempt to obtain a cultural definition of health and diabetes from American Indian women. The pervasiveness of diabetes was readily apparent: most participants had at least one family member or friend with diabetes. Although one third of the participants had diabetes, the responses received from those without diabetes mirrored the responses of those with diabetes. An analysis of responses stratified by diagnosis of diabetes would provide little additional information.

Many factors — historical, political, sociocultural, and geographical — impact health perceptions among American Indians (19). Challenges abound in trying to define health as American Indians perceive it, especially through the lens of Western medicine. A gap exists between the discernment of a biologically defined chronic disease and the more culturally relevant presence of physical symptoms; this gap presents a strong barrier to accurate assessment of personal health status (18,22,23). In one study of DinÃ© (Navajo) families with asthmatic children, asthma is perceived by the families as a series of individual episodic reactions requiring attention instead of an underlying physiological chronic inflammatory condition (23); the findings of the DinÃ© study agree with our findings. Hatton reported that elderly American Indians define their health in terms of the absence or presence of various chronic diseases (19). These results also concur with perceptions found in our sample. Also in the Hatton report, the capacity of individuals to perform activities of daily living and take care of themselves was regarded as an important aspect of personal health assessment (19); this capacity was deemed important by our study participants as well.

Of particular interest was the mutual dependency of the cultural definitions of health and diabetes. The women in our study held the belief that being unhealthy was discernable by physically feeling ill. Interviews with older American Indians residing in urban areas considered themselves to be healthy in the absence of any outward, perceivable sign of illness (19). The disjointed impression among our respondents that long-term complications are symptoms of disease (instead of the consequences of poor diabetes control) may be explained by the perception that one is not unhealthy unless a perceptible feeling of illness is present. When our study participants faced a clinical diagnosis of diabetes, they delayed self-care until long-term complications — accompanied by a decrease in physical function — became evident. To these women, long-term complications serve as the only tangible evidence of illness. It is this strong reliance on physical symptomology that provides a great obstacle to diabetes prevention, screening, and care.

A strong sense of inevitability pervaded the many ideas surrounding the pursuit of health and prevention of diabetes among our American Indian sample. Many, but not all, participants believed that diabetes is inevitable and ultimately leads to death, especially for individuals who have strong family histories of the disease. Previous research with the Gila River Indian Community describes these feelings of inevitability as "fatalistic" attitudes that moderate the perception of diabetes prevention and may serve as additional barriers to adopting prevention behaviors (16). Kozak reported an overall sense of surrender to diabetes, which was viewed as an inevitable, uncontrollable disease that resulted in death (16). Additionally, Judkins reported "highly fatalistic attitudes and verbalizations" about diabetes among the Seneca, accompanied by a feeling of powerlessness against the disease (17). It has been theorized that fatalism has developed as a social coping mechanism to deal with the severity of the diabetes epidemic and the resulting compromised quality of life (16). Compensatory mechanisms built into cultural personality to deal with environmental and personal stress may precipitate denial or avoidance behaviors (17). A sense of inevitability may ultimately result in a decreased propensity to take necessary steps for disease prevention, which is often misconstrued by the administrators of Western medicine as non-compliance (16,18).

Additional barriers were evident in the prevention and treatment of diabetes in these American Indian women. Family dynamics play a critical role in health care in American Indians (22). With a shift from traditional economic strategies to mainstream business practices, traditional American Indian families are shifting toward more Western nuclear families, which has an impact on family dynamics (22). Additionally, family resistance to alterations in dietary habits serves as an additional barrier to diabetes prevention and care in American Indian women. To achieve successful behavior change, nutrition education and diabetes prevention programming must involve the family unit. To what extent will family obligations or positive social support structures within Native American communities allow self-care behaviors? How receptive are American Indian individuals to external support, and what is their capacity to overcome barriers for health promotion? These questions — as yet unanswered — require more research.

In addition to conflicts between healthy lifestyle behaviors and family obligations, avoidance of diabetes screening serves as an additional barrier to diabetes treatment. The women expressed an inclination to avoid screening even if they harbored suspicions of having the disease. Many diagnoses were reported while women were seeking medical treatment for unrelated reasons. If a clinical diagnosis is made, denial is likely, especially when no physical symptoms are apparent. Similarly, Huttlinger et al reported a case of a Diné woman who was taken to the doctor for a routine check-up against her will and subsequently diagnosed with diabetes (18). Though vehemently claiming she felt fine, she had to undergo amputation because of the serious progression of her uncontrolled diabetes. This case demonstrates the family's role in encouraging women to seek care and how the lack of the physical signs of disease can hinder treatment.

There are several limitations of the current study. First, American Indian women were hired from local American Indian communities to conduct the interviews, regardless of previous experience in qualitative interviewing techniques. Despite training in such techniques, they varied in the amount they probed interview participants on topics important to the research team. To address this concern, transcript reviewers analyzed interview tapes soon after retrieval and provided feedback to interviewers as additional training and guidance. Second, because transcript reviewers functioned as the research instrument, the lens through which reviewers read the transcripts provided bias. To address this concern, transcripts were read by the 2 researchers independently from each other and then discussed until consensus was reached on coding. We achieved an inter-rater reliability of more than 90%. Furthermore, the lack of responses related to traditional healing practices and the role of spirituality may have been due to the recruitment of participants through tribal health clinics and is not likely representative of all American Indian cultures. Though snowball sampling aided in recruitment, participants recruited from the health clinics may have been more likely to seek medical treatment through the health clinics than through traditional healing practices. Finally, the sample was derived through non-probability methods. Though these methods may decrease the generalizability of the findings, they are often needed to identify individuals from an at-risk population (19).

Despite these limitations, the congruency of the data to other reports of perceptions of diabetes among other American Indian groups provides support for our findings (19). Though results similar to ours have been reported, they were derived from reservation-living American Indian groups; we have identified perceptions of health and diabetes among a sample population outside the reservation setting. Our findings indicate a more comprehensive approach to the underlying issues in health promotion and diabetes prevention than previous reports. Previous reports did not address the interrelationships of perceptions of health nor did they discuss issues of diabetes prevention. We have attempted to address some of these issues; however, each new issue presents new unanswered questions, indicating a need for further investigation of the cultural definitions of health and diabetes.

Efforts to identify disparities in health perceptions and worldviews are essential for developing nutrition education interventions that precipitate behavior change (24-26). Previous research and multi-site programs, including *Awakening the Spirit: Pathways to Diabetes Prevention & Control* (American Diabetes Association) and the National Diabetes Prevention Program (National Institute of Diabetes, Digestive and Kidney Diseases), have demonstrated improved diabetes prevention and treatment by targeting specific lifestyle behaviors within the context of American Indian communities (27-29). American Indian communities vary widely in tribal affiliation and location; future researchers must identify the characteristics of each American Indian population studied to ensure they meet the community's specific needs (30). The importance of solid formative data on a population is paramount, especially considering that a large portion of research is conducted on reservations. Furthermore, the extent to which the perceptions held by American Indian women in Northeast Oklahoma are congruent with other American Indians within and outside of Oklahoma needs to be examined to assist in designing effective education programs.

## Figures and Tables

**Table 1:** *Cultural Structure of Health and Diabetes*: Questioning Guide for Interviewing Oklahoma American Indian Women about Cultural Perceptions of Health and Diabetes, 2003

1.	Describe your current health to me.
2.	Describe how you feel about your health.
3.	What, if any, health concerns do you have?
4.	What are the major health concerns of other Indian women you know?
5.	What do you think is the leading cause of death for Indian women in the United States?
6.	What comes to mind when I mention diabetes?
7.	Let's discuss diabetes a bit. What do you think causes a person to get diabetes? Why do you think these things (mentioned above) cause diabetes? If eating right, describe how people should eat. What keeps people from eating right? If exercise, what should they do and how often? What keeps people from getting exercise? How did you find the information that you just told me?
8.	What do you think happens to a woman once she develops diabetes?
9.	Can you think of anyone who is at risk for developing diabetes? (Is he or she Indian?)
10.	How can a person tell if he or she has diabetes? How do they feel?
11.	Tell me about anything that you know of that might keep a woman from developing diabetes. Why do you think these things (mentioned above) prevent diabetes? Where did you find this information? If read, where? Books, magazines (which ones)? If heard, where? From whom?
12.	What may prevent a woman from doing the things that may prevent diabetes?
13.	What treatments are there for diabetes that you know about? If diet, describe the diet.
14.	Who are you concerned about developing diabetes? What are the reasons that you are concerned about this person(s)?
15.	What can parents or family do to help prevent this person/child from developing diabetes?
16.	What can the tribe or community do to help prevent this person/child from developing diabetes?
17.	How do you feel about diabetes?
18.	What is your greatest fear about diabetes?
19.	What control do you think a person has over diabetes?
20.	Can you prevent diabetes? When can a person begin to do these things to prevent diabetes?
21.	How would you describe a traditional (Indian) diet (the old way of eating)? What would you think of shifting the diet back toward the old ways Indians used to eat? Do you think eating a more traditional diet would help Indians prevent diabetes?
22.	Is there anything else would you like to tell me about diabetes?

**Table 2:** Parent Code Words Used in Initial Coding of Verbatim Transcripts of Interviews of Oklahoma American Indian Women About Cultural Perceptions of Health and Diabetes, 2003

**Code Word**	**Definition**
AT RISK	Characteristics of those at-risk for developing diabetes
AVOIDANCE	Avoiding screening or seeking treatment for ailment for fear of diabetes diagnosis
AWARENESS	Lack of or increased awareness of diabetes
BAR DM	Barriers in controlling diabetes
BAR EXER	Perceived barriers preventing exercise
BAR FOOD	Perceived barrier to eating a healthy diet
CONTROL	Methods used to control diabetes
DEF DIET	Cultural definition of a healthy diet
DEF DM	Cultural definition of diabetes
DEF EXER	Cultural definition of exercise
DEF HEALTH	Cultural definition of health
DENIAL	Denial experienced post-diagnosis of diabetes
DM CAUSES	Perceived causes of diabetes
DM CONCERN	Concerns and fear about diabetes
DM DIET	Perceived diabetic diet and dietary changes required by diabetes diagnosis
DM LT COMP	Perceived long-term complications of diabetes
DM PREVENT	Methods to prevent or delay onset of diabetes
DM SYMPTOM	Perceived symptoms of diabetes onset
DM TREAT	Perceived treatments for diabetes
FAMILY	Role of family in diabetes prevention and treatment
HEALTHCARE	Issues in quality and continuity of health care
MEN HEALTH	Perceptions of men and health
NA DEATH	Perceived leading causes of death for American Indian women
NA WOMEN	Perceived health issues of other American Indian women
PERSONAL	Personal health concerns
SOCIAL	Social aspects of diabetes care and prevention
SOURCE	Sources of health and nutrition knowledge
TRAD DIET	Cultural definition of a "traditional diet"
TRIBE	Role of tribe/community in diabetes prevention and treatment

**Table 3:** Demographic Characteristics of Oklahoma American Indian Women Interviewed About Cultural Perceptions of Health and Diabetes, 2003

**Characteristic^a^**		
Age (years)	43.4 ± 11.4	
Degree of Indian blood (%)	64.6 ± 0.3	
Body mass index^b^(kg/m^2^)	32.1 ± 6.9	

	**N**	**%**

**Marital status** c		
Married	30	38.5
Not married	48	61.5
**Education**		
High school or less	24	7.6
Some college	44	55.7
College degree	11	13.9
**Employment**		
Employed	52	65.8
Not employed	27	34.2
**Annual household income** c		
< $15,000	36	46.2
$15,000-$24,000	20	25.6
>;$25,000	22	27.2
**BMI categories (kg/m** ^2^ **)^b^,^c^**		
Healthy (18.5-24.9)	9	12.2
Overweight (25.0-29.9)	21	28.4
Obese (>30)	44	59.5
**Diagnosed Diabetes^c^**		
Yes	26	32.9
No	50	63.3

a Presented as mean ± SD.

b Based on self-reported height and weight.

c Does not sum to n = 79 due to missing data.
